# A Normal Range of KL-6/MUC1 Independent of Elevated SP-D Indicates a Better Prognosis in the Patients with Honeycombing on High-Resolution Computed Tomography

**DOI:** 10.1155/2011/806014

**Published:** 2010-12-28

**Authors:** Shu Hisata, Yuichiro Kimura, Naoko Shibata, Shuichi Ono, Takao Kobayashi, Shigeki Chiba, Hiromitsu Ohta, Toshihiro Nukiwa, Masahito Ebina

**Affiliations:** ^1^Department of Respiratory Medicine, Tohoku University Graduate School of Medicine, 1-1 Seiryo, Aoba-ku, Sendai 980-8574, Japan; ^2^Department of Radiology, Hirosaki University Graduate School of Medicine, Hirosaki 036-8562, Japan; ^3^Department of Respiratory Medicine, Sendai Kosei Hospital, Sendai 980-0873, Japan

## Abstract

Both SP-D and KL-6/MUC1 are established biomarkers of the interstitial pneumonias, including idiopathic pulmonary fibrosis (IPF), but the causes and clinical outcomes based on their independent effects are not known. Eleven asymptomatic patients, detected with honeycombing on high-resolution computed tomography (HRCT), were compared with 17 other IPF outpatients having slight respiratory symptoms and honeycombing as well. Although SP-D was increased in both groups, KL-6 was significantly higher in the symptomatic IPF group. When the patients (*n* = 11) having both biomarkers elevated were compared with the other patients (*n* = 6) with only SP-D elevated, the distribution of fibrotic lesions with honeycombing on HRCT was larger and the survival time was shorter in the patients having both biomarkers elevated. Immunohistochemical analysis also differentiated these biomarkers in the lung. These results suggest both a cause and the prognostic value of dissociation of these biomarkers.

## 1. Introduction

Although the prognosis for patients with idiopathic pulmonary fibrosis (IPF) is very poor after a diagnosis of respiratory symptoms, that is, a median survival of 2–4 yrs [1], the natural history of IPF, from the point of view of asymptomatic status, is not well understood. According to the American Thoracic Society/European Respiratory Society (ATS/ERS) Consensus Statement in 2002, “respiratory symptoms and abnormalities on pulmonary function test are required for the diagnosis of IPF in the absence of surgical biopsy” [2]. Therefore, most of the patients with IPF reported previously seem to have been of advanced stage. However, the detection of early stage of asymptomatic patients with IPF is important for the understanding the pathogenesis of this progressive pulmonary fibrosis, and for developing more effective treatments of IPF. 

Several studies have reported patients in early stages of interstitial lung disease (ILD) [3–6]. Arakawa et al. followed dust-exposed patients and reported that honeycombing required a median of 12 years to develop in normal or near-normal lungs [3]. Rosas et al. evaluated family members of patients with familial pulmonary fibrosis to identify asymptomatic patients and suggested that progression from early asymptomatic to symptomatic status takes place over a period of decades [5]. Although radiological findings on high-resolution computed tomography (HRCT) has been reported [3, 6], there is only limited information on the relevant biomarkers, such as surfactant protein D (SP-D) and Krebs von den Lungen-6 Antigen/MUC1 (KL-6/MUC1) in early stages of IPF [4]. 

SP-D and KL-6/MUC1 are useful biomarkers in the diagnosis of various ILDs, such as IPF, collagen vascular disease-associated interstitial pneumonitis, radiation pneumonitis, hypersensitivity pneumonitis, pulmonary alveolar proteinosis, and drug-induced pneumonitis [7, 8]. SP-D belongs to the collectin subgroup of the C-type lectin superfamily [9]. Clara cells, nonciliated epithelial cells in the peripheral airways, and alveolar type II epithelial cells are known to secrete the protein in the airspace [7]. KL-6/MUC1 is a circulating high-molecular-weight glycoprotein, classified as human MUC1 mucin [10]. MUC1 is expressed on the surface membrane of alveolar type II epithelial cells and bronchiolar epithelial cells [11]. Most patients with ILD display elevated SP-D and KL-6/MUC1 in parallel, which is considered to be due to the fact that both are derived from regenerated alveolar type II epithelial cells in interstitial pneumonias [8, 12]. Although some patients reportedly exhibit a divergence between SP-D and KL-6/MUC1 [13], the mechanism and the clinical value of each biomarker has not been clarified yet.

The initial aim of this study was to reveal the characteristics of the biomarkers SP-D and KL-6/MUC1 in early-stage IPF. In 2002, we found out that asymptomatic patients with honeycombing on HRCT have elevated SP-D but not KL-6/MUC1 in their serum. Therefore, as second aim, to evaluate the prognostic value of the divergence between SP-D and KL-6/MUC1, we surveyed the outcome of these patients for 8–10 years. We also performed an immunohistochemical study to confirm the dissociation of these biomarkers in the lung tissues obtained by surgical lung biopsy. 

## 2. Material and Methods

### 2.1. Patients

We conducted a retrospective study of 28 outpatients at Tohoku University Hospital and Sendai Kousei Hospital, Sendai, Japan, between 1999–2001. All patients met radiological findings of definite usual interstitial pneumonia (UIP) pattern on HRCT, (1) subpleural, basal predominance, (2) reticular abnormality, (3) honeycombing with or without traction bronchiectasis, and (4) absence of features listed as inconsistent with UIP pattern. In addition, none of these patients had any evident disease, such as collagen diseases or hypersensitivity pneumonia, reportedly causative of ILD. Bacteriological examinations were of no clinical significance in all patients. In the 28 patients, two groups, asymptomatic (*n* = 11) and symptomatic patients (*N* = 17), were included. Asymptomatic patients (*n* = 11) had no respiratory symptoms, and normal pulmonary function. Therefore, these patients could not be diagnosed as having IPF on the basis of ATS/ERS 2002 consensus classification statement [2], but could be in the early stage of IPF. In asymptomatic patients, some abnormalities on chest X-ray had been detected in the course of the health examination system in Japan, which was subsequently confirmed to meet the HRCT criteria of UIP pattern. In contrast, the symptomatic patients (*n* = 17) had certain respiratory symptoms, such as cough or dyspnea on exertion, but less than Hugh-Johns level II, at most. They were diagnosed as having IPF on the basis of 2002 consensus classification statement (clinical *n* = 15, surgical *n* = 2) [2]. Next, we extracted two groups, SP-D (*n* = 6) and SP-D&KL-6 group (*n* = 11), based on the SP-D and KL-6/MUC1 serum levels (Table 2) from these 28 patients. The SP-D group was defined as the patients with a serum SP-D more than two times the cut off level (110 ng/ml), and serum KL-6/MUC1 less than the cut off level (500 U/ml), which for SP-D > 220 ng/ml and KL-6/MUC1 < 500 U/ml. SP-D&KL-6 group was defined as the patients with serum SP-D and KL-6/MUC1 more than two times the cut off levels (SP-D > 220 ng/ml and KL-6/MUC1 > 1000 U/ml).

### 2.2. Survey of Outcome of the Patients

We surveyed the outcome of the patients by telephone call or postal mail in August 2009. We could not contact one patients of each in SP-D and SP-D*＆*KL-6 group.

### 2.3. Pulmonary Function Tests

Measurements of vital capacity (VC), FVC, FEV_1_, ratio of FEV_1_ to FVC (FEV_1.0_%), total lung capacity (TLC), ratio of residual volume (RV) to TLC (RV/TLC), and the diffusing capacity of the lung for carbon monoxide (DL_CO_) were made using standard equipment (CHESTAC-55V; CHEST Co., Tokyo, Japan) according to the ATS recommendations [14]. 

### 2.4. Collection and Analysis of Blood Samples

Peripheral venous blood samples were collected between 1999–2001. Each serum sample was analyzed for SP-D and KL-6/MUC1. The serum level of SP-D was measured by a commercially available enzyme-linked immunosorbent assay (ELISA) kit (Yamasa Co., Japan), as previously described [15]. Serum KL-6/MUC1 was measured by a commercially available sandwich ELISA kit (Eisai Co., Japan) [10]. The serum cut-off levels were 110 ng/ml (SP-D) and 500 U/ml (KL-6/MUC1).

### 2.5. Analysis of HRCT Scan Imaging

All patients first underwent conventional CT scanning of the chest using 10-mm thick sections. The HRCT scans also were performed within two weeks of the collection of the blood sample. The findings on HRCT were evaluated by discussion among the authors, including a radiologist. On the basis of previous study results [16], we measured the distribution of fibrotic lesions with honeycombing in three levels (aortic arch, carina, and the top of diaphragm) on HRCT images for each patient using a computerized image-analyzer system (NIH image).

### 2.6. Immunohistochemistry for SP-D and KL-6

Specimens of the three IPF lung tissues were obtained by video-assisted thoracoscopic surgery at Tohoku University Hospital. Lung specimens were fixed in 10% buffered formalin for 18 h and embedded in paraffin for immunohistochemistry. The antibody against KL-6 was provided by Sanko Junyaku Co., Ltd. (Tokyo, Japan) and SP-D by YAMASA (Tokyo, Japan); both of which are commonly used for detecting the serum levels of these biomarkers. An antihuman podoplanin monoclonal antibody (AngioBio Co., Del Mar, CA) was used for detecting lymphatic endothelial cells, and antibodies against human CD34 (Nichirei) and von Willebrand factor (vWF) (Nichirei) were used for endothelial cells in the lung. The antigen-antibody complex was visualized using Vector Red (Vector Laboratories, Burlingame, CA) and/or diaminobenzidine (DAB), and counterstained with elastica-Goldner stain, modified elastica-Mason stain. For immunofluorescent staining, the Alexa Fluor Dye (Molecular Probes, Invitrogen) appropriate for each antibody was used according to the manufacturer's instructions.

### 2.7. Statistical Analysis

For comparison between asymptomatic and symptomatic patients (Table 1), SP-D and SP-D&KL-6 group (Table 2), data were analyzed by Mann-Whitney nonparametric test. For comparison of SP-D and KL-6/MUC1 between asymptomatic and symptomatic patients, data were analyzed by paired *t*-test. Differences of the distribution of fibrotic lesions with honeycombing in SP-D and SP-D&KL-6 group were compared with the use of paired *t*-test. The survival duration was estimated using the Kaplan-Meier method, and comparison was made using the log rank test. In all tests, *P *values <  .05 were considered statistically significant.

## 3. Results

### 3.1. Serum SP-D Is Elevated Even in Asymptomatic Patients with Honeycombing on HRCT

The clinical characteristics and pulmonary function data are summarized in Table 1. The age of the asymptomatic patients ranged from 49–80 yrs, and that of the symptomatic patients ranged from 46–76 yrs. In the pulmonary function data, the DL_CO_ was lower in the symptomatic IPF patients than that in the asymptomatic patients. 

**Figure 1:**
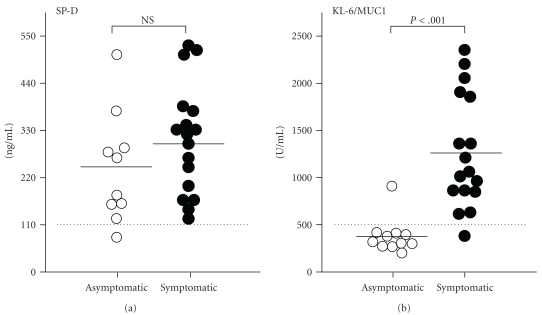
Comparison of serum levels of SP-D and KL-6/MUC1 between asymptomatic patients with honeycombing and symptomatic IPF patients. Both groups have elevated SP-D without statistic difference, but KL-6/MUC1 was significantly higher in symptomatic IPF patients. Dotted lines represent mean values of SP-D (110 ng/ml) and KL-6/MUC-1 (500 U/ml).

The level of serum SP-D and KL-6/MUC1 was compared among asymptomatic and symptomatic IPF patients (Figure 1). The level of serum SP-D was elevated in both asymptomatic and symptomatic IPF patients (235.8 ± 41.0, 310.7 ± 31.1 ng/ml, resp.). The levels of serum KL-6/MUC1 in asymptomatic and symptomatic IPF patients were 423.9 ± 61.0, 1256.2 ± 143.5 U/ml, respectively. Although the mean level of KL-6/MUC1 in asymptomatic patients was higher in comparison with those in normal volunteers (258 ± 131 U/ml) reported in previous study [17], most of the asymptomatic patients had a normal range of KL-6/MUC1.

### 3.2. Patients with Elevated SP-D Independent of KL-6 Had Limited Fibrotic Lesions with Honeycombing on HRCT

To analyze the clinical value of SP-D and KL-6/MUC1, we extracted two groups from these 28 patients by the elevated biomarkers, an SP-D group (*n* = 6) and SP-D&KL-6 (*n* = 11) group, because none of these patients had high KL-6/MUC1 with a normal range of SP-D. The SP-D group consisted of five asymptomatic patients and one symptomatic patient. The SP-D&KL-6 group consisted of eleven patients, all symptomatic. The clinical characteristics and pulmonary function data of these patients are summarized in Table 2. The age of the patients in the SP-D group ranged from 66–80 yrs, and in the SP-D&KL-6 group ranged from 49–74 yrs. In the pulmonary function data, vital capacity was lower in the SP-D&KL-6 group than the SP-D group. 

To analyze the relation of biomarkers and fibrotic lesions with honeycombing on HRCT, we measured the distributional area of fibrotic lesions with honeycombing in three levels of the HRCT of each patient (Upper; aortic arch, Middle; carina, Lower; top of diaphragm). The mean percentage of the area of fibrotic lesions with honeycombing was significantly lower in the SP-D group than the SP-D&KL-6 group Table 2.

### 3.3. The Patients with Only an Elevated SP-D Level Had Longer Survival

**Figure 2:**
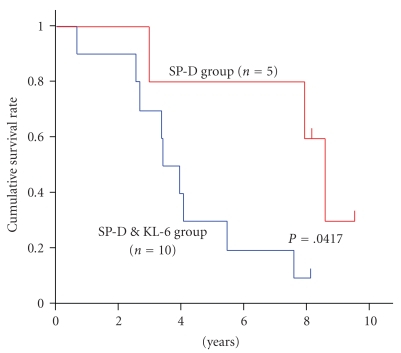
The Kaplan-Meier survival curves of patients with honeycombing in SP-D and SP-D&KL-6 groups. Patients in SP-D group (in red) had longer survival (mean survival time, MST; 3117 days) than those in SP-D&KL-6 group (in blue) (MST; 1232 days) (*P* = .0417).

We surveyed the clinical outcome of patients in the SP-D group and the SP-D&KL-6 group for 8–10 years after initial detection. Because we could not contact one patient in each group, we compared the overall survival between the SP-D only group (*n* = 5) and the SP-D&KL-6 group (*n* = 10) by Kaplan-Meier analysis (Figure 2). Two of five patients in the SP-D group died of pneumonia. One patient died of unknown cause. Two patients were alive with slight respiratory symptoms. In contrast, four of ten patients in the SP-D&KL-6 group died of acute exacerbation of IPF; one patient died of progression of chronic respiratory failure, one patient died of pneumonia, one patient died of pancreas cancer, and two patients died of unknown causes. One patient is still alive.

### 3.4. Distribution of SP-D and KL-6/MUC1 in IPF Lungs

**Figure 3:**
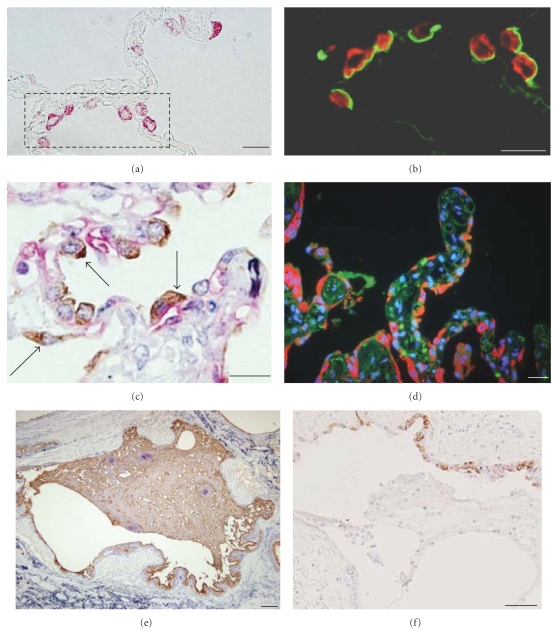
Immunohistochemical distribution of SP-D and KL-6/MUC1 in control and IPF lungs. ((a) and (b)) In control lungs, alveolar type II epithelial cells show cytoplasmic staining for SP-D (in red). In contrast, KL-6/MUC1 (in green, (b)) distributes extracellular surface facing alveolar space without cytoplasmic or basal-lateral side expression. The dotted square in (a) is the same section with (b) observed by fluorescence (scale bar = 10 *μ*m). (c) Double immunohistochemistry of SP-D (in brown) and CD34-positive alveolar capillary endothelial cells (in red) in IPF lung tissues. The alveolar type II epithelial cells with SP-D expression are always in close contact with alveolar capillaries (indicated by arrows). Counter staining by elastic-Goldner staining (scale bar = 10 *μ*m). (d) In IPF lung tissues, regenerated type II epithelial cells are increased with more intense expression of SP-D (in red) than control lungs. These increased type II epithelial are also in close contact with alveolar capillaries (in green). Nuclei are stained in blue (scale bar = 10 *μ*m). ((e), (f)) In honeycomb cysts in IPF lings, thick mucus with immunoreactive for KL-6/MUC1 (in brown, (e)) were accumulated in contrast to SP-D (in brown, (f)). Counterstained by elastica-Goldner staining (scale bars = 100 *μ*m).

**Figure 4:**
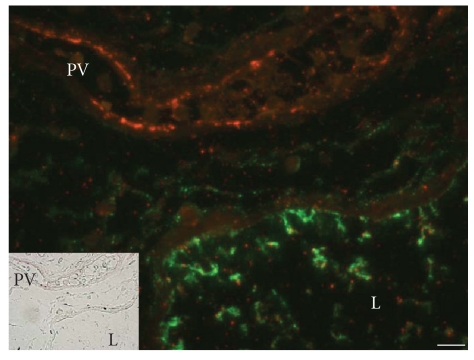
Immunohistochemistry of SP-D and KL-6/MUC1 in IPF lungs. Double-fluorescent immunohistochemistry for SP-D (in red) and KL-6/MUC1 (in green) in IPF lung tissues revealed SP-D-positive materials within both pulmonary veins (PV) and lymphatics (L). In contrast, KL-6/MUC1 was not included in pulmonary veins but only in lymphatics (scale bars = 10 *μ*m).

There is a clear difference in the immunohistochemical distribution pattern between SP-D and KL-6/MUC1. While both of the biomarkers are produced by alveolar type II cells and bronchiolar epithelial cells, the immunoreactivity for SP-D is cytoplasmic, while KL-6 is distributed at extracellular sites facing the alveolar space or airway space (Figures 3(a) and 3(b)). In IPF lung tissues, regenerated type II epithelial cells are increased in number and immunoreactive intensity of SP-D of these cells are also increased (Figures 3(c) and 3(d)). Most of these epithelial cells are in close contact with alveolar capillaries (Figures 3(c) and 3(d)). In honeycomb lesions, KL-6/MUC1 is more broadly distributed inside the cystic space than SP-D (Figures 3(e) and 3(f)). Interestingly, both KL-6 and SP-D are observed in the lymphatics, but KL-6 was not distributed in the pulmonary veins, where only SP-D was included (Figure 4).

## 4. Discussion

It was shown here that (1) the serum level of KL-6/MUC1 was maintained within the normal range in dissociation with the SP-D level, which was elevated in asymptomatic patients with honeycombing on HRCT, (2) these patients with high SP-D but low KL-6 in their serum exhibited prolonged survival, (3) there are obvious differences in the distribution patterns between SP-D and KL-6/MUC1 in the IPF lung, especially in the clearance route, which may cause the observed dissociation between these biomarkers.

The finding of honeycombing on HRCT is believed to be mostly associated with usual interstitial pneumonia (UIP), as it is reported that honeycombing in at least one lobe indicate UIP, with a 90% sensitivity and 86% specificity [18]. In this study, we enrolled the asymptomatic patients with honeycombing in the subpleural and basal lungs as having early-stage-IPF. In these patients, the serum level of SP-D was significantly high, even though the KL-6 level stayed within the normal range. These results indicate that serum SP-D is a more sensitive biomarker than serum KL-6/MUC1 to detect early IPF and has also been reported to be able to detect the early changes of ILD in patients with systematic sclerosis [19].

Our survey of the survival of these patients clearly noted that patients with a high level of SP-D but low KL-6 in their serum had a better prognosis. Several studies have showed the prognostic value of the biomarkers, SP-A, SP-D, and KL-6 at the initial visit [7, 10, 20, 21], but no studies have referred the clinical outcomes related to the dissociation of the serum levels of SP-D and KL-6. Our results indicate that KL-6 is a better prognostic marker than SP-D in IPF patients.

The different mechanistic roles of the biomarkers SP-D and KL-6 are explained in part from the immunohistochemical study results, which revealed the different distribution patterns of these biomarkers in IPF lungs (Supplement Figure 1). KL-6 is expressed at the extracellular surface of alveolar type II cells and bronchiolar epithelial cells, and from these cells is released into the alveolar lining fluid, accumulated in honeycomb cysts. Since KL-6 is a high molecular weight mucin-like glycoprotein (molecular weight > 1,000 KD) [17], our results showing KL-6 is found in the lymphatics, but not in the pulmonary veins, may be reasonable in that KL-6 is too large to be cleared by the alveolar capillaries, and is cleared by the lymphatics only [22]. In contrast, SP-D is distributed in the cytoplasm of these cells, and SP-D is found in both the pulmonary lymphatics and pulmonary veins. Because SP-D is a very small molecule (43 KD), consisting of four trimeric subunits [23], SP-D or its fragments may be absorbed from alveolar capillary endothelial cells, which are always in close contact with alveolar type II epithelial cells [24]. In addition, because the interlobular lymphatics chiefly working for alveolar clearance is reduced in IPF lungs [22], it may be reasonable that the KL-6/MUC1 in the alveolar space is delayed to be cleared and delivered to serum of IPF patients. These findings are thought to explain why SP-D is elevated in asymptomatic patients and that KL-6 is elevated in the later stages.

There are several limitations in our study. First, the number of subjects in this study is low. And we did not evaluate dyspnea quantification by means of scales, such as MRC, in initial subjects enrollment. Second, we could not analyze changes of lung function from baseline, because of retrospective study. Therefore, we could not evaluate regression analysis between biomarkers concentration and lung functional changes from baseline.

In conclusion, SP-D and KL-6 are useful biomarkers for differentiating patients with honeycombing on HRCT into the stable and progressive stages, which would inform clinical implication for IPF.

## Figures and Tables

**Table 1:** Characteristics of asymptomatic and symptomatic patients with honeycombing on HRCT.

	Asymptomatic	Symptomatic	*P* values
Patients (M/F)	10/1	15/2	
Age yrs	67 ± 3 (*n* = 11)	66 ± 2 (*n* = 17)	.32
Smoking Current/Ex/Never	5/2/1	5/4/5	.44
Pack-yrs	33.3 ± 6.3 (*n* = 8)	33.1 ± 7.6 (*n* = 14)	.60
VC %pred	96.9 ± 6.1 (*n* = 9)	88.1 ± 3.6 (*n* = 15)	.25
FEV_1.0_/FVC (%)	82.3 ± 3.4 (*n* = 9)	84 ± 2.2 (*n* = 15)	.61
DL_CO_ %pred	79.3 ± 6.1 (*n* = 6)	58.3 ± 5.8 (*n* = 14)	.031*
RV/TLC %	32.6 ± 1.8 (*n* = 5)	34.5 ± 2.0 (*n* = 15)	.36

Data are presented as mean ± S.E. VC: vital capacity. EFV_1.0_: forced expiratory volume in one second. FEV: forced expiratory volume. DL_CO_: diffusing capacity of lung for carbon monoxide. RV/TLC: Residual volume/Total lung capacity. *: <.05.

**Table 2:** Characteristics of patients in SP-D and SP-D&KL-6 group.

	SP-D group	SP-D&KL-6 group	*P* values
Age yrs	71 ± 2.2 (*n* = 6)	66 ± 2.1 (*n* = 11)	.19
Pack-yrs	28.6 ± 11.5 (*n* = 4)	31.0 ± 12.2 (*n* = 8)	.72
VC %pred	102.4 ± 7.3 (*n* = 5)	82.5 ± 4.5 (*n* = 9)	.045*
FEV_1.0_/FVC (%)	82.9 ± 5.1 (*n* = 5)	87.3 ± 2.1 (*n* = 9)	.69
DL_CO_ %pred	71.6 ± 16.5 (*n* = 3)	61.6 ± 7.8 (*n* = 9)	.051
RV/TLC (%)	34.9 ± 4.1 (*n* = 3)	32.9 ± 1.7 (*n* = 9)	.40
SP-D ng/ml	336 ± 40.3 (*n* = 6)	375 ± 30.8 (*n* = 11)	.48
KL-6/MUC1 U/ml	393 ± 20.5 (*n* = 6)	1439 ± 169 (*n* = 11)	<.001*
HRCT scan fibrotic area (%)			
Aortic arch	0.7 ± 0.2 (*n* = 6)	14.9 ± 4.7 (*n* = 11)	<.001*
Carina	1.1 ± 0.5 (*n* = 6)	15.8 ± 5.6 (*n* = 11)	<.001*
Top of diaphragm	5.4 ± 2.4 (*n* = 6)	33.4 ± 7.6 (*n* = 11)	<.001*

Data are presented as mean ± S.E. SP-D group: SP-D > 220 ng/ml and KL-6 < 500 U/ml; SP-D&KL-6 group: SP-D > 220 ng/ml and KL-6 > 1000 U/ml. *: *P* < .05.
